# Tunable Intracavity Coherent Up‐Conversion with Giant Nonlinearity in a Polar Fluidic Medium

**DOI:** 10.1002/advs.202405227

**Published:** 2024-07-22

**Authors:** Daichi Okada, Hiroya Nishikawa, Fumito Araoka

**Affiliations:** ^1^ Center for Emergent Matter Science (CEMS) RIKEN 2‐1 Hirosawa Wako Saitama 351‐0198 Japan

**Keywords:** nonlinear optical response, optical microcavity, polar nematic liquid crystal, second‐harmonic generation, up‐conversion

## Abstract

The study has demonstrated a novel microcavity‐based flexible photon up‐conversion system using second harmonic generation (SHG) from a polar nematic fluidic medium doped with a laser dye. The idea is based on coherent light generation via stimulated emission (lasing) and simultaneous frequency doubling inside a microcavity. The polar nematic fluid equips very high even‐order optical nonlinearity due to its polar symmetry and large dipole moment along the molecular long axis. At the same time, its inherent fluidic nature allows to easily functionalize the media just by doping, in the present case, with an emissive laser dye. The demonstrated system exhibits a giant nonlinear optical response to input light, while enabling spectral narrowing and multiple‐signal output of up‐converted light, which is not attainable through the simple SH‐conversion of input light. Furthermore, the susceptibility of the liquid crystal offers dynamic modulation capabilities by an external stimulus, such as signal switching by the application of electric field or wavelength tuning through temperature variation. Such a brand‐new type of simple coherent flexible up‐conversion system must be promising as a new principle for easily accessible and down‐scalable wavelength conversion devices.

## Graphical abstract

The study has developed a novel microcavity‐based coherent up‐conversion system. This system provides a multitude of advantages, such as giant nonlinearity, increased efficiency, spectrum narrowing, and the generation of coherent multimode outputs, which are usually difficult to achieve in conventional SHG. Furthermore, the outputting signal can be dynamically modulated by external stimuli.

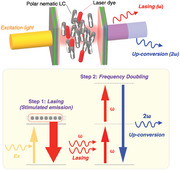

## 1 Introduction

Photon up‐conversion, a process for producing higher‐energy photons from lower‐energy photons, is not only useful for wavelength conversion in laser systems, but is also a candidate technology for boosting the efficiency of important photo‐processes in photovoltaics, photocatalysis, and optogenetics. Second‐harmonic generation (SHG), a well‐known fundamental photon up‐conversion process, is based on the optical nonlinearity exhibited in symmetry‐broken materials, which is ubiquitous and, therefore, utilized in various applications, such as bioimaging, phototherapy, optical communications, and optical computing. However, the intrinsic conversion efficiency of SHG is generally so small that it is necessary to introduce an extrinsic mechanism to intensify the light–matter interactions, for example by phase matching,^[^ ^1^ ^]^ a photonic effect,^[^ ^2^ ^]^ or confinement in resonators such as Fabry–Pérot (FP) etalons, micro/nano‐spheres/disks, or plasmonic nanostructures.^[^ ^3^ ^]^ Of those, one of the most common approaches involves the use of an optical microcavity structure as a convenient and effective method for obtaining an adequate conversion efficiency in SHG. Since the photons are densely confined in a small volume with a size comparable to the optical wavelength, light–matter interaction can be strongly enhanced to provide an SHG that is one to three orders of magnitude stronger.^[^ ^3^ ^]^

**Figure 1:**
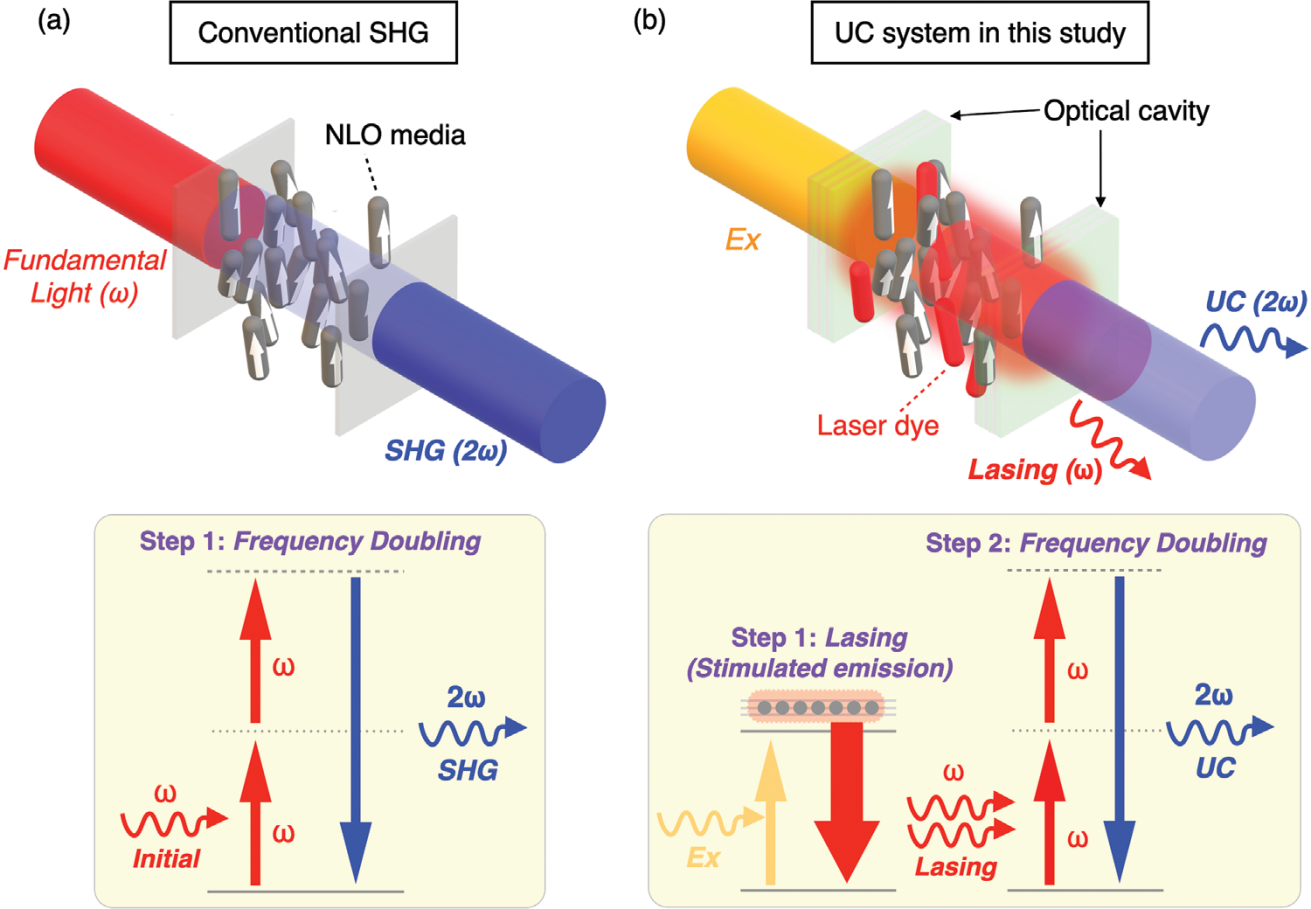
a) Conventional up‐conversion scheme of SHG. The propagating incident fundamental light photons are directly converted into frequency‐doubled SHG photons. b) The up‐conversion (UC) system demonstrated in this study, which involves a simultaneous stimulated emission (lasing) and an SHG process. Here, the incident laser is used for the excitation of the fluorescent material inside the cavity.

Here, we demonstrate a novel type of photon up‐conversion using an FP microcavity, in which a dye‐doped polar nematic liquid crystal (PNLC) permits two‐step intracavity coherent light generation through stimulated emission (lasing) with simultaneous frequency doubling (**Figure** **1**). The main role of the PNLC is wavelength conversion by means of its excellent nonlinear optical property originating from its polar symmetry and its large dipole moment along the long molecular axis. At the same time, the inherent fluidic nature of the PNLC allows it to achieve an optical gain merely by doping with a laser dye. Another advantage of the system is its ease of preparation. Because an FP cavity structure is essentially the same as that of a sandwich‐type liquid‐crystal (LC) cell, the LC material can be easily introduced into a FP cavity. As an optical device, this system readily improves the monochromaticity of the fundamental light and the efficiency of the resultant SHG, in addition to displaying a giant nonlinear optical response to incident light. Multimode signal output is also achievable, depending on the cavity conditions. Such advantageous characteristics are not easily attainable in a conventional SHG system. Furthermore, the flexibility of the LC provides our optical device with switchability and tunability in response to external fields. Our coherent up‐conversion system, which is achieved by both stimulated emission and SH conversion, holds promise for future applications in information‐processing devices based on nonlinear optics.

## 2 Results and Discussion

**Figure 2:**
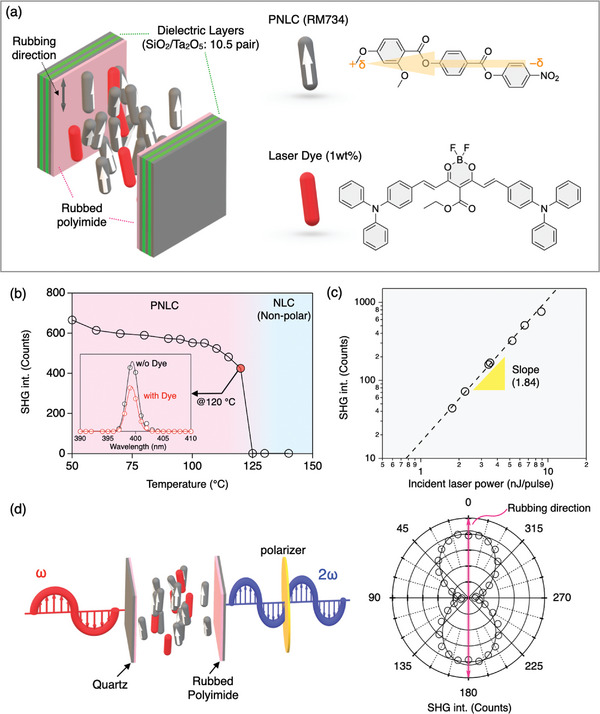
a) Schematic representation of the optical cavity, and the molecular structures of the PNLC (RM734) and the fluorescent dye. b) Temperature dependence of the SHG intensity from the PNLC. SHG activity is observed below 120 °C, the temperature for the phase transition to the ferroelectric nematic state. The inset figure shows SHG spectra at 120 °C for the undoped PNLC (red) and the PNLC doped with the fluorescent dye (black). c) SHG intensity as a function of the incident laser intensity. d) Polar plot of the polarization‐angle dependence of the SHG signal.

To provide proof of our concept, we used a recently developed ferroelectric nematic liquid crystal as our PNLC medium.^[^ ^4^ ^]^ Although LC materials of this kind have a highly fluidic nature, they still exhibit an extremely strong SHG, signifying a remarkable nonlinear optical property.^[^ ^4d^ ^]^ Meanwhile, doping with a small molecule is an effective way to functionalize an LC material; for example, mixing an LC and a photoisomerizable azobenzene produces a photoinducible athermal phase transition,^[^ ^4^, ^5^ ^]^ whereas simple doping of a cholesteric liquid crystal with an organic laser dye makes possible cavity‐less lasing.^[^ ^6^ ^]^ Indeed, this kind of approach also works for a PNLC, and several examples have already been demonstrated in which additional functional properties are combined with ferroelectricity to realize unprecedented applications, such as photovariable capacitors,^[^ ^4h^ ^]^ tunable polar cholesteric reflectors,^[^ ^4c,g^ ^]^ electrically controllable microlenses,^[^ ^4o^ ^]^ and an enhanced nonlinear optical response.^[^ ^4k^ ^]^ We therefore hit on the idea of a flexible coherent up‐conversion system that uses a doped PNLC as a functionalized nonlinear optical medium. Here, we used one of the most famous PNLC material, RM734, which has already known to exhibit great SH conversion efficiency (≈70 times higher than that of a quartz reference) simply by injecting it into an LC cell.^[^ ^4d^ ^]^ The PNLC was doped with a boron difluoride curcuminoid‐based fluorescent dye (1 wt.%) (**Figure** **2**a), which is capable of lasing in the near‐infrared range (700–800 nm).^[^ ^7^ ^]^ Because the primary absorption band of the PNLC is in the UV region below 350 nm, the self‐absorption effect was negligible in any of our results (Figure S1, Supporting Information]. The doped PNLC was injected into a microcavity structure consisting of two quartz substrates separated by a 5 µm spacer (Figure S2, Supporting Information), the inner surfaces of which were equipped with dielectric reflectors (%*T* <0.5% in the range 700–800 nm) (Figure S3, Supporting Information). To obtain a uniform planar orientation, rubbed polyimide was used in the alignment layers. The unidirectional alignment was confirmed by polarized optical microscopy (Figure S4, Supporting Information). Our home‐built optical setup is shown schematically in Figure S5 (Supporting Information). Upon irradiation of the fundamental/excitation laser pulses (≈200 fs, 10 kHz) from an optical parametric amplifier, the output light was spectroscopically recorded by an optical multi‐channel analyzer (OMA), and simultaneously, its intensity was measured by a photon‐counter attached to a monochromator.

First, we examined the conventional transmission SHG from the doped PNLC introduced into a standard LC cell without reflectors. Heating to its isotropic temperature of 180 °C, the sample was gently cooled while the output light was monitored. After undergoing a phase transition to a ferroelectric nematic LC phase at ≈120 °C, a strong monochromatic light peak appeared at the half wavelength (≈400 nm) of the fundamental light (≈800 nm) (Figure 2b). Hereafter, the temperature was fixed at 120 °C, unless otherwise noted. The intensity of the resulting signal increased nearly quadratically with fundamental light intensity (Figure 2c), clearly evidencing the SHG, even in the doped PNLC. The self‐absorption effect of the doped dye led to a slight reduction of SHG compared to that of RM734 itself (Figure 2b, inset). This effect could also explain the slightly lower logarithmic slope (1.84) observed, in contrast to the ideal value of 2.0. We scanned the fundamental light wavelength of incident laser in the possible lasing range of the dye (from 700 to 800 nm) and we found that there was an average loss of SHG of ≈30% in comparison to that of the undoped PNLC (Figure S6, Supporting Information). Such a reduction might arise from the self‐absorption effect and/or a slight disordering in the doped PNLC. However, the output SHG is highly linearly polarized to the rubbed direction (Figure 2d), so that the disorder effect should be minor.

**Figure 3:**
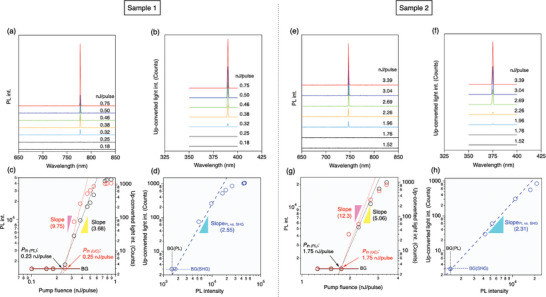
a,b,e,f) The PL and up‐converted light spectra at various excitation intensities for two different samples in which lasing peaks appeared at (a) 777 nm (Sample 1) and (e) 747 nm (Sample 2), respectively. b,f) The up‐converted light peaks appeared at the half wavelengths of the lasing peaks. c,g) The PL and up‐converted light intensities plotted as functions of the excitation (incident laser) intensities. The estimated thresholds [*P*_th (PL)_, *P*_th (UC)_] are indicated, together with their logarithmic slopes. d,h) The up‐converted light intensity plotted as a function of the PL (lasing) intensity.

Next, we investigated the lasing and up‐conversion properties of a dye‐doped PNLC confined in the microcavity structure. To excite the laser dye, the irradiated laser wavelength was tuned to 600 nm, close to the maximum value of its absorption peak. The beam spot was focused to ≈100 µm by a lens. **Figure** **3**a,e show two photoluminescence (PL) spectra for two different cavity conditions (Samples 1 and 2), originating from the deviation of the refractive index caused by a slight disorder in the alignment of the liquid crystalline media or an inhomogeneity in the cavity length (cell thickness). As increasing the excitation intensity, narrow lasing peaks appeared at 777 nm (Sample 1) and 747 nm (Sample 2), and their intensities increased with obvious nonlinearity (Figure 3c,g; black dotted line). The difference in the lasing wavelengths of the two samples is due to the difference in the cavity conditions, as discussed above. The lasing threshold, estimated from the intersection of the PL and background (BG) slopes in the logarithmic plot (Figure 3c,g), was 0.23 nJ/pulse for Sample 1 and 1.75 nJ/pulse for Sample 2; their nonlinearity below the saturation was evaluated from the logarithmic slopes to be 3.68 and 5.06, respectively (the black dotted lines in Figure 3c,g). The difference in the threshold stems mainly from the difference in optical gain. Specifically, 777 nm is distant from the absorption region of the dye, so that the lower self‐absorption in this region leads to a better optical gain than that observed at 747 nm.

In addition to lasing, a sharp peak appeared in the near‐ultraviolet (UV) region, at almost half the wavelength of the lasing peak discussed above (Figure 3b,f). This additional emission peak had almost the same excitation threshold as that of the lasing, and no emission could be detected below this threshold. In addition, this UV emission peak was observed only when the PNLC was in ferroelectric nematic phase showing SHG activity (Figure S7, Supporting Information). This peak is considered to correspond to originates from the simultaneous SH conversion of the lasing emission, that is, coherent up‐converted light. This up‐converted light intensity was also nonlinearly dependent on the excitation intensity (the red dotted line in Figure 3c,g), but its power law appears to have been different from that of lasing. As estimated from Figure 3c,g, the logarithmic slopes for these two conditions are 9.75 and 12.30, respectively, which are much larger than those of the lasing itself and typical SHG (Figure 2c). This giant nonlinearity is attributed to the combined nonlinearities of the simultaneous lasing and SHG processes in the present up‐conversion system. A plot of the up‐converted intensity versus the lasing intensity shows the presence of almost quadratic dependencies, with logarithmic slopes of 2.55 and 2.31, as shown in Figure 3d,h, respectively. Of course, if this was the usual SHG, the logarithmic slope should be just 2.0 or slightly less, as in the case of Figure 2c. However, the slightly larger logarithmic slope shows the presence of a higher efficiency than that of the usual conversion. Because the action of the lasing light as the fundamental light is a result of its confinement in the cavity (unlike the SHG), we need to know the actual intensity of the lasing light field in the cavity to fully understand the present up‐conversion behavior. If another effect, such as a photorefractive effect or a photobleaching effect, is present, the situation becomes more complicated. The former might cause power‐dependent phase matching/unmatching between the lasing and SHG lights, which may change the efficiency. In any case, the situation is too complicated to be analyzed, and a full explanation will remain a problem for future study.

The system also enables us to modulate the up‐conversion efficiency merely by changing the optical conditions in the cavity. It is known that, in many cases, anisotropic dye molecules, when embedded in an LC medium, align to the nematic director uniaxially along their molecular long axes. The transition dipole moment of the dye is then also aligned, and this makes lasing most efficient when the excitation light is linearly polarized in the same direction of alignment of the LC. In the present case, the nonlinear polarization is also directed along the director of the PNLC; in other words, the up‐conversion efficiency is modulated by the polarization state. Likewise, the output up‐converted light is also linearly polarized along the alignment direction (Figure S8, Supporting Information). The estimated dichroic ratio is ≈0.78, so that the conversion efficiency is lower if the sample is excited with an excitation light orthogonal to the direction of alignment.

**Figure 4:**
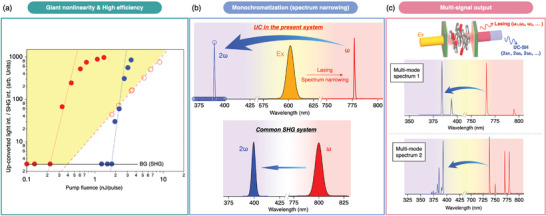
a) Comparison of the intracavity up‐conversion (Sample1, Sample2) and the conventional SHG (reprises of Figures 3c,g, and 2d, respectively). The up‐conversion process shows much more significant nonlinearity. The conversion efficiency is improved up to about two orders of magnitude higher than the conventional SHG process when the lasing threshold is lower (red filled circles in (a)). b) Schematics of spectral narrowing in the present up‐conversion process mediated by the stimulated emission. c) Multimode coherent up‐conversion.

**Figure** **4**a compares the power laws in the present up‐conversion system (600 nm → 390 nm) and the usual SHG (800 nm→400 nm). Due to the high slope efficiency, it is evident that the former overtakes the latter in the high‐intensity region. However, due to the saturation of the optical pumping, the up‐conversion intensity also saturates at high pump fluences, whereas the SHG does not do so. Another notable feature is the narrow bandwidth of the up‐converted spectrum. Generally, the character of the output SHG is determined by that of the fundamental light. For example, a broadband fundamental light of ultra‐short pulses (typically, from a femtosecond laser) results in broadband SHG. However, although the present up‐conversion is based on SHG, output with a narrower bandwidth (more monochromatic) is always obtained, regardless of the bandwidth of the irradiated excitation light. This is because the present up‐converted light is generated through intracavity SH conversion of the lasing, the bandwidth of which is typically determined by the characteristics of the microcavity and the laser dye (Figure 4b).

Regarding the above, we introduce an interesting feature: multimode coherent up‐conversion. Because the lasing condition is influenced by the optical characteristic of the microcavity, as mentioned above, we can realize multimode lasing by introducing spatial inhomogeneity into the system. Here, we demonstrate two examples of multimode lasing (the red curves in Figure 4c), both of which are observed within the same PNLC device but at different positions. Accordingly, the resultant up‐converted light from each position also becomes multimodal (the blue curves in Figure 4c), giving a coherent up‐converted light with multiple wavelength peaks despite the use of a single FP device and a single excitation light of 600 nm.

**Figure 5:**
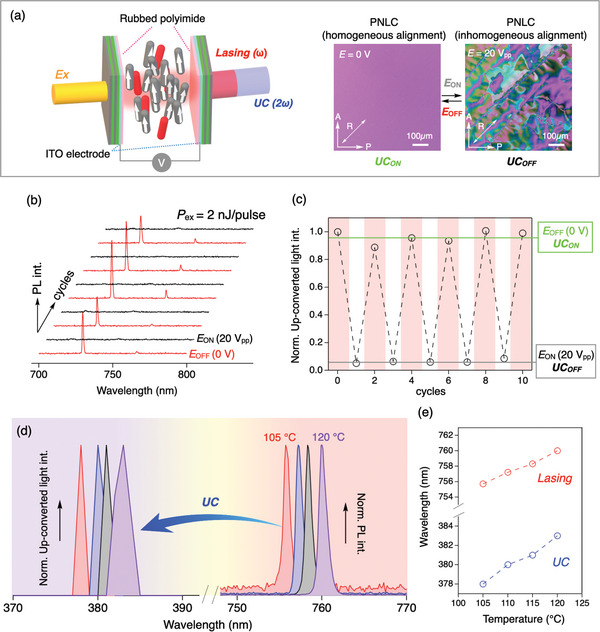
a) Schematic representation of the F–P microcavity with transparent electrodes (ITO), and polarized microscopy images in the presence and absence of an ac electrical voltage of 20 V_pp_ at 200 Hz. b,c) ON/OFF switching of (b) lasing and (c) up‐converted light on changing the electric field. d) Variation of lasing and up‐conversion spectra upon cooling. e) The temperature dependence of the peak top wavelengths.

Finally, we demonstrate stimuli‐responsive modulation in the presence of an electric field or on changing the temperature. To apply an electric field, we fabricated an FP microcavity device with dielectric reflectors, the tops of which were coated with transparent indium–tin oxide (ITO) electrodes, as shown in **Figure** **5**a. Initially, a uniform texture was observed by polarized‐light microscopy. Upon applying an AC electric field perpendicular to the substrate (20 V_pp_, 200 Hz), dynamic and chaotic turbulence occurred, disrupting the ordered structure of the PNLC (Figure 5a). As a result, the lasing threshold increased markedly, leading eventually to deactivation (Figure 5b,c; Figure S9, Supporting Information). Thus, the up‐conversion can be switched off by turning on an electric field. When the electric field is turned off, alignment is restored and the up‐conversion reappears. In this way, we can switch the up‐conversion on or off at will by the application of an electric field (Figure 5a–c). In addition, by heating or cooling, it is also possible to tune the wavelength of the up‐converted light through the continuous changes in the cavity conditions arising from temperature‐dependent alterations in the refractive index and/or thermal expansion/shrinkage. As decreasing the temperature within the ferroelectric nematic phase from 120 to 105 °C, the lasing wavelength was continuously blue‐shifted, and, accordingly, the up‐conversion wavelength was also blue‐shifted (Figure 5d,e). Temperature‐tunable SHG systems have been achieved in various structures, including disks, waveguides, and fibers.^[^ ^8^ ^]^ In these systems, the tunable wavelength range is wider than our demonstrated system, however, “tunable” refers to the ability to adjust phase matching conditions, allowing the wavelength at which the most efficient SHG occurs to be tuned by temperature. Thus, to achieve the desired SHG wavelength, it is necessary to change the wavelength of the fundamental light source. In contrast, our demonstrated system does not require changing the fundamental light source to tune the SHG wavelength; it only requires exciting the dye. This is beneficial for developing down‐scalable tunable NLO devices. Furthermore, by leveraging the phase transition from the ferroelectric nematic phase to the nematic phase through temperature control, it is possible to achieve SHG‐only switching while maintaining lasing emission (Figure S7, Supporting Information). These unprecedented results further demonstrate the unique properties of our flexible coherent up‐conversion system.

## 3 Conclusion

In conclusion, we have developed a novel intracavity coherent up‐conversion system based on a Fabry–Pérot microcavity filled with a dye‐doped PNLC. The system is quite simple, yet provides a multitude of advantages, such as a giant nonlinearity, increased efficiency, spectrum narrowing, and the generation of coherent multimode outputs, which are usually difficult to achieve in conventional SHG. In addition, because our system is inherently soft due to the presence of liquid crystallinity, the resulting signal can be dynamically modulated by external stimuli. In our study, the PNLC does not satisfy either phase‐matching or quasi‐phase‐matching conditions. However, recent advances in the study of PNLCs have shown that phase‐matching or quasi‐phase‐matching can be attained by tuning the helical pitch of a cholesteric structure in PNLCs through the doping of chiral molecules, resulting in efficient SHG.^[^ ^2g^ ^]^ By potentially combining this approach with our system, we can anticipate achieving much more efficient intracavity SH conversion system. Furthermore, if the more efficient NLO is attained, since our system can generate multi‐mode coherent light signal inside single optical cavity, the more widely tunable coherent up‐conversion would be possible by utilizing nonlinear wave mixing such as sum‐frequency generation.^[^ ^9^ ^]^ Our demonstrated flexible microcavity‐based coherent up‐conversion system extends the usefulness of soft ferroelectrics and could lead to the development of innovative nonlinear optical devices in the future.

## Conflict of Interest

The authors declare no conflict of interest.

## Supporting information

Supporting Information

## Data Availability

The data that support the findings of this study are available from the corresponding author upon reasonable request.
