# Supplementary material for: Tunable Intracavity Coherent Up‐Conversion with Giant Nonlinearity in a Polar Fluidic Medium
Source: Adv Sci (Weinh). 2024 Jul 22;11(36):2405227. doi: 10.1002/advs.202405227 (PMC11423090; doi:10.1002/advs.202405227)
Supplement: Supplementary file 1 — Supporting Information [file ADVS-11-2405227-s001.pdf]

ADVANCED SCIENCE
Open Access

Supporting Information

for *Adv. Sci.*, DOI 10.1002/advs.202405227

Tunable Intracavity Coherent Up-Conversion with Giant Nonlinearity in a Polar Fluidic Medium

Daichi Okada, Hiroya Nishikawa and Fumito Araoka**

Supporting Information

Daichi Okada*, Hiroya Nishikawa, Fumito Araoka*

Table of Contents

1. Materials and Instruments --- 2

2. Linear optical response and sample preparation --3

3. Optical setup -- 5

4. SHG and UC---6

1. Materials and Instruments

Unless otherwise noted, all reagents and solvents were used as received. The ferroelectric nematic liquid crystal (RM734) was purchased from Instec., Inc, USA. The used fluorescent dye was a commercially available boron difluoride curcuminoid derivative from Luminescence Technology Corp., Taiwan. The alignment material (AL1254) was a product of JSR Corp., Japan.

UV-VIS absorption and transmission spectra were recorded using a JASCO V-770 spectrophotometer. The dielectric mirror of alternating layers of 10.5 pairs of SiO_2 and Ta_2O_5 was fabricated using a magnetron sputtering system (SRV4320, Shinko-Seiki, Japan).

2. Linear optical response and sample preparation

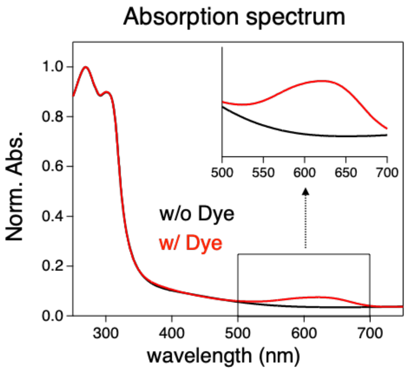

Figure S1, Absorption spectra of RM734 and a dye-doped RM734 thin films spin-coated on a quartz substrate.

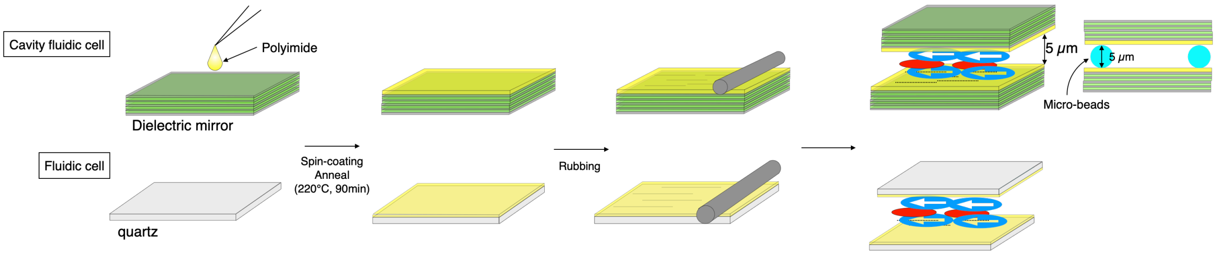

Figure S2 Schematization of the fabrication process of the F-P microcavity device based on a LC cell.

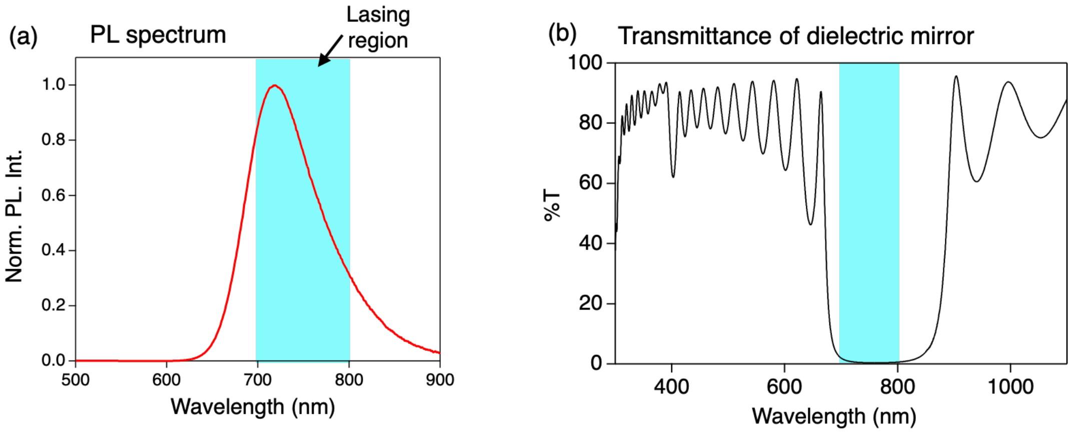

Figure S3, (a) The PL spectrum of the dye-doped PNLC; (b) Transmission of the dielectric mirror whose reflection band is adjusted to the PL peak wavelength (indicated by a light blue box), where the lasing action is highly probable.

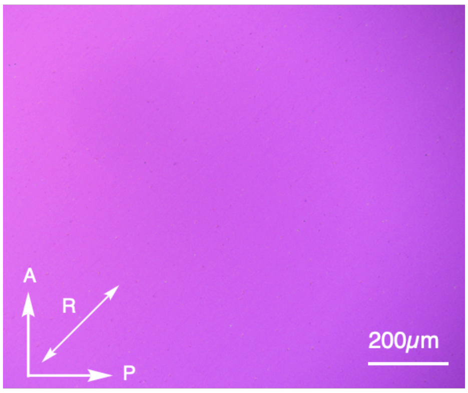

Figure S4, A polarized microscope image of the planarly-aligned dye-doped PNLC in a LC cell at 120°C.

3, Optical setup

Our optical setup is illustrated in Fig. S5. We mainly used two laser sources: one at 800 nm from regenerative amplifier and the other at 600nm from an optical parametric amplifier with a regenerative amplifier (OPA9400 with RegA, Coherent Inc., USA), both operating at a frequency of 10 kHz and with a pulse width of ~200 fs. The 600 nm pulse was used to excite the fluorescent dye, and the 800 nm is used for typical SHG investigation. The sample was heated in a hot stage with a temperature controller (HSC402 with mk2000, Instec Inc., USA). The obtained signal was collected by an objective lens (Mitsutoyo Corp., Japan) in the transmission direction. The PL spectra were recorded using an optical multichannel analyser (OMA) (USB4000, Ocean Optics Inc., USA), while SHG was detected with a photon-counting head (H7421, Hamamatsu Photonics K.K., Japan) on a monochromator. The SHG spectra were recorded in steps of 1 nm. Reference SHG spectra measured using these two spectroscopic setups were compared, and we confirmed that there is no need for calibration between them.

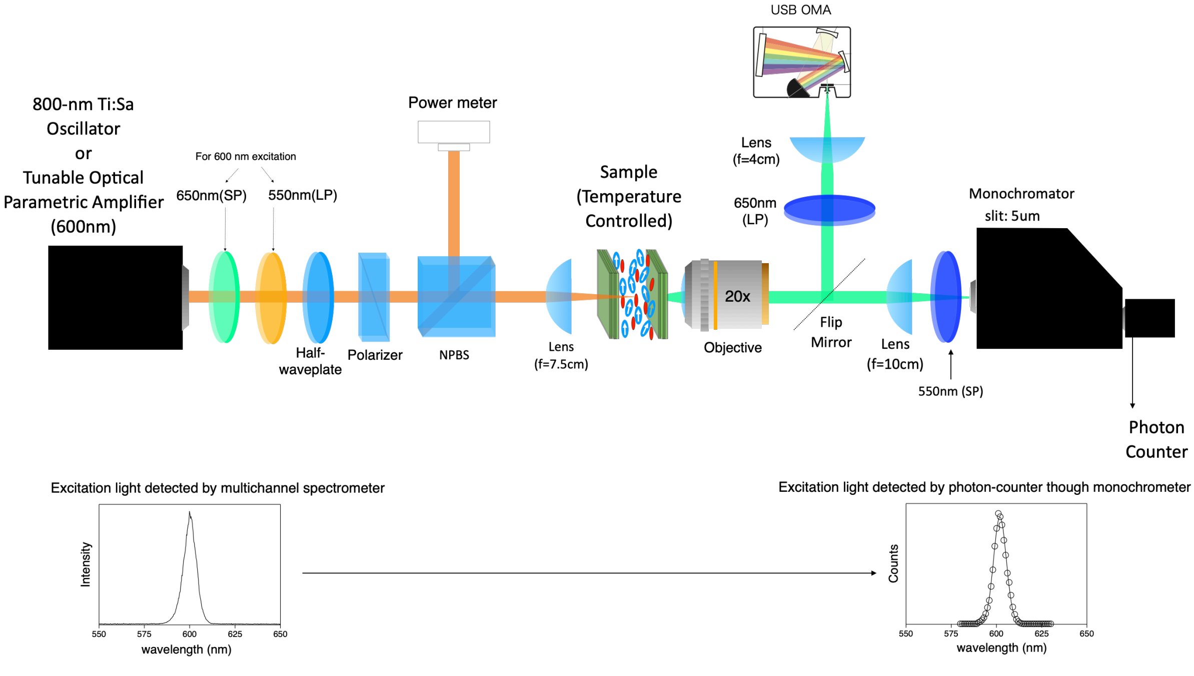

Figure S5, Our homemade optical setup for PL and SHG spectroscopy.

4, SHG and UC

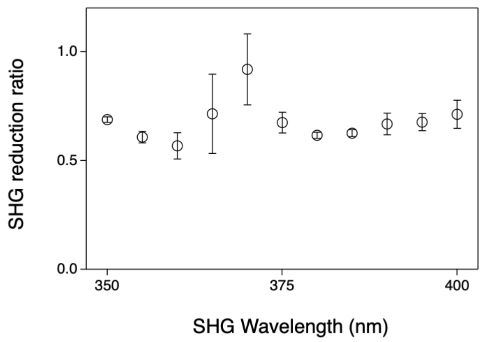

Figure S6, Influence of dye doping on SHG as a reduction ratio depending on the wavelength. The fundamental laser wavelength was scanned from 800 nm to 700 nm in steps of 10 nm, the reduction ratio was defined as a ratio of SHG intensities from the samples with and without dye-doping.

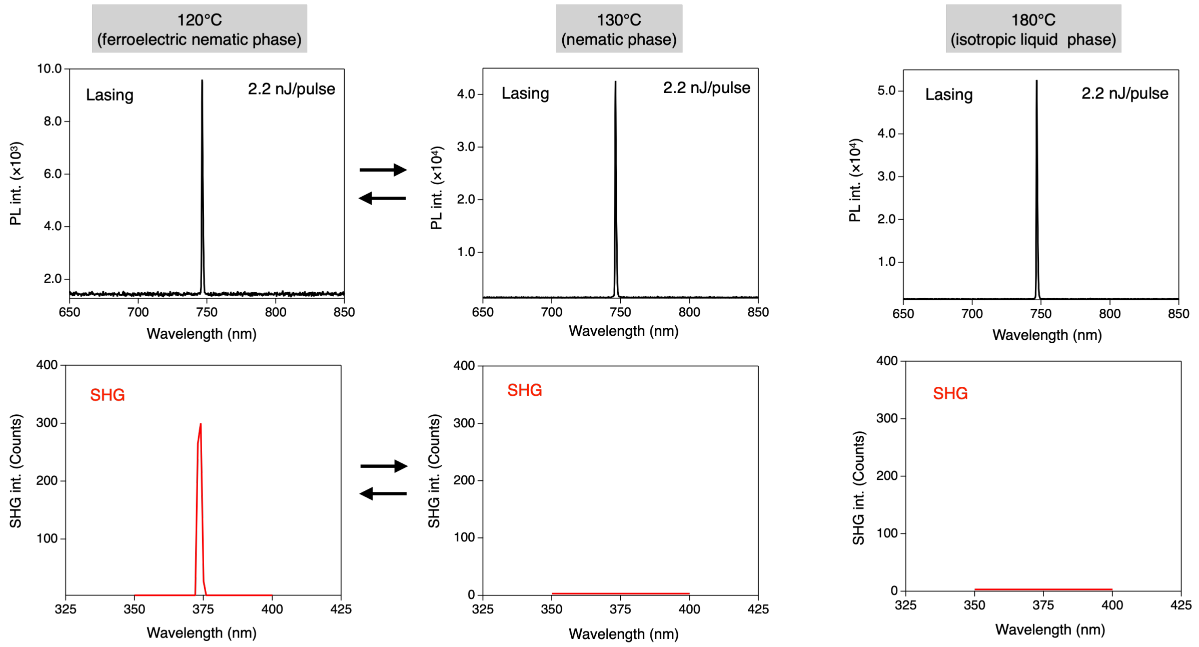

Figure S7, Lasing and SHG spectra at 120°C, 130°C and 180°C. Each temperature corresponds to ferroelectric nematic phase, nematic phase and isotropic liquid phase, respectively. PNLC exhibits SHG activity only in the ferroelectric nematic phase, therefore, any up-converted UV emission is not observed at 130°C and 180°C. In other words, it is possible to realize SHG-only switching by temperature variation, while maintaining lasing emission.

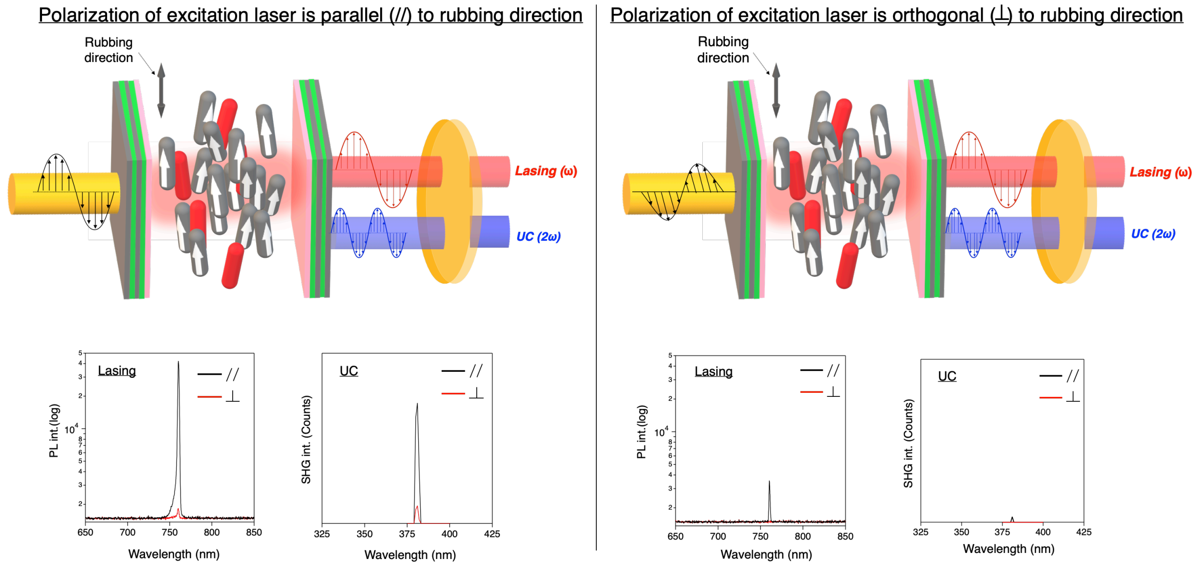

Figure S8, The polarization dependency of output optical signal under the different excitation polarization conditions.

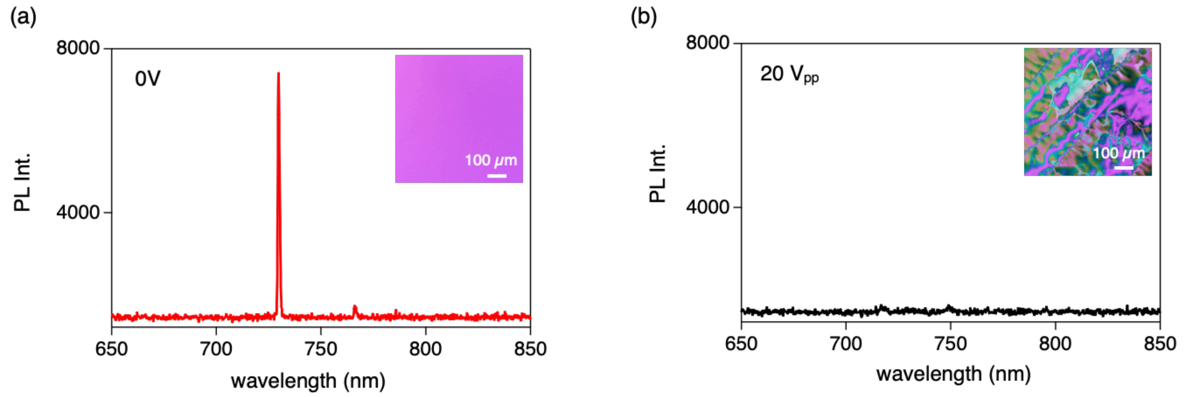

Figure S9, ON/OFF behavior of lasing (a) without and (b) with applying an electric field.
